# Assessment of choroidal structural changes in patients with pre- and early-stage clinical diabetic retinopathy using wide-field SS-OCTA

**DOI:** 10.3389/fendo.2022.1036625

**Published:** 2023-01-19

**Authors:** Fabao Xu, Zhiwen Li, Xueying Yang, Yang Gao, Zhiwei Li, Guihua Li, Shaopeng Wang, Xiaolin Ning, Jianqiao Li

**Affiliations:** ^1^ Department of Ophthalmology, Qilu Hospital, Shandong University, Jinan, China; ^2^ Shandong Key Laboratory: Magnetic Field-free Medicine & Functional Imaging, Jinan, China; ^3^ Research Institute of Shandong University: Magnetic Field-free Medicine & Functional Imaging, Jinan, China; ^4^ School of Physics, Beihang University, Beijing, China; ^5^ Jinan Aier Eye Hospital, Jinan, China; ^6^ Zibo Central Hospital, Binzhou Medical University, Zibo, Shandong, China

**Keywords:** diabetes mellitus, diabetic retinopathy, wide-field optical coherence tomographic angiography, choroidal vascular changes, choroidal vascularity index (CVI)

## Abstract

**Purpose:**

To investigate the micro-vascular changes in choroidal structures in patients with pre- and early-stage clinical diabetic retinopathy (DR) using wide-field Swept-Source Optical Coherence Tomography Angiography (SS-OCTA).

**Method:**

This observational cross-sectional study included 131 eyes of 68 subjects that were divided into healthy controls (group 1, n = 46), pre-DR (group 2, n = 43), early-stage DR (group 3, n = 42) cohorts. All participants that underwent SS-OCTA examination were inpatients in the department of Ophthalmology and the department of Endocrinology, Qilu Hospital, Shandong University, and Department of Ophthalmology, Aier Eye Hospital, Jinan, from July 11, 2021 to March 17, 2022. The choroidal vascularity index (CVI), choroidal thickness (ChT) and central macular thickness (CMT) in the whole area (diameter of 12 mm) and concentric rings with different ranges (0–3, 3–6, 6–9, and 9–12 mm) were recorded and analyzed from the OCTA image.

**Result:**

Compared with healthy eyes, decreases in CVI and ChT were found in the eyes of patients with pre-or early-stage DR. The changes were more significant in the peripheral choroid, with the most prominent abnormalities in the 9-12mm area (P < 0.001). However, there was no obvious difference in the average CMT value. Furthermore, CVI and ChT were significantly correlated with the duration of diabetes in the range of 6–9 and 9–12 mm (Ps < 0.05; Correlation coefficient = -0.549, -0.395, respectively), with the strongest correlation (Ps < 0.01; Correlation coefficient = -0.597, -0.413, respectively) observed at 9–12 mm.

**Conclusion:**

The CVI and ChT values of diabetic patients are significantly lower than in healthy controls, especially in patients with early-stage DR. In addition, the peripheral choroidal capillaries are more susceptible to early DM-induced injury than in the central area.

## Introduction

Current evidence suggests that the annual prevalence of diabetes mellitus (DM) has increased in recent years (1, 2). According to the latest Diabetes Atlas (10^th^ edition) released by the International Diabetes Federation (IDF) in 2021, about 537 million adults aged 20-79 suffer from DM worldwide (https://diabetesatlas.org/). Diabetic retinopathy (DR) is widely acknowledged as one of DM’s most serious ocular complications and the most common cause of blindness (3–6). Given the lack of obvious symptoms in early-stage disease, patients are often diagnosed in the vision-threatening stage of DR, when irreversible damage to visual acuity (VA) has already occurred. Therefore, effective early screening and intervention are essential to prevent severe vision loss secondary to DR. An increasing body of evidence suggests that a sustained hyperglycemic environment can cause vascular endothelial cell dysfunction and narrowing of the choroidal and retinal capillaries (7, 8). As the permeability of the inner retinal blood barrier increases, retinal exudates hemorrhage and edema occur, leading to irreversible damage to the retinal photoreceptors (9, 10). There are reportedly no retinal capillaries in the fovea within a diameter of 0.5 mm, and the choroidal circulation is the only blood supply for this area. In addition, the ellipsoid zone of the outer retina is supplied by choroidal circulation (11–13). Accordingly, the assessment of choroidal capillaries parameters can potentially detect retinal damage in early-stage disease and reveal the pathological mechanism of DR. Since Saracco and colleagues first put forward the conception of diabetic choroidopathy in 1982, clinical studies on choroids have constantly been emerging, and our understanding of diabetic choroidopathy has gradually deepened (14–16). As a new biological measurement tool, the choroidal vascularity index (CVI) has been documented to provide a more stable and objective assessment of choroidal conditions by measuring the ratio of the choroidal vessels to tissue composition (17). Current evidence suggests that CVI and choroidal thickness (ChT) enable quantitative analysis of the morphological and structural changes of choroidal circulation abnormalities and can be used as early assessment indicators for many chorioretinal diseases (8, 18, 19).

Optical coherence tomographic angiography (OCTA) can effectively assist in investigating the quantitative characteristics of changes in choroidal microvascular circulation and is the main imaging modality for the early detection and evaluation of diabetic choroidopathy (20–22). Given that the traditional OCTA (3 mm × 3 mm or 6 mm ×6 mm) has a limited visual field, the pathological features of the peripheral retina of DM patients cannot be obtained (22–24). Wide-field swept source (SS)-OCTA represents a new non-invasive instrument suitable for various follow-up applications. Importantly, with a scanning scope enlarged to 12×12 mm, wide-field SS-OCTA enables more comprehensive observation of diseases (25, 26). In this study, we sought to measure CVI and the thickness of the retina and choroid with wide-field SS-OCTA to better understand vascular abnormalities in early-stage DM patients and promote the early detection and treatment of diabetic retinopathy.

### Methods

This retrospective observational study was conducted as per the tenets of the Declaration of Helsinki and approved by the Medical Research and Ethics Committee of Qilu Hospital and Shierming Hospital. Written informed consent was obtained from subjects after explaining the purpose of the study and informing them of the details and any potential risks involved in the study. The study included 46 eyes of healthy controls and 85 eyes of patients with DM. All subjects were treated in the Department of Ophthalmology and Department of endocrinology, Qilu Hospital, Shandong University, and Department of Ophthalmology, Aier Eye Hospital, Jinan, from July 11, 2021 to March 17, 2022.

In this study, we first shortlisted patients with type 2 DM and the healthy control group subjects were matched for age, gender and refractive error distribution. Subjects were divided into the following three groups according to ophthalmoscopy and fundus photography (Patients were grouped according to the eye with more advanced lesions): Group 1, healthy subjects without diabetes; Group 2, pre-DR patients (Diabetes patients without DR); Group 3, diabetic patients with early-stage DR (DR patients with only microaneurysm). The exclusion criteria were as follows: (1) age ≥ 18 years old; (2) Subjects with serious systemic diseases (tumor, stroke, dementia, etc.); (3) Subjects with diabetic macular edema; (4) History of ocular trauma and other vitreoretinal surgery; (5) Subjects with glaucoma, high myopia (spherical equivalent >= −6.00D), and other ocular conditions that may affect the choroidal and retinal capillaries; (6) Ocular media opacity; (7) Images of low quality (quality index < 6).

The three groups of subjects underwent standardized examinations. Ophthalmic examinations included best-corrected visual acuity (BCVA) and slit lamp bio-microscopy. Demographic data of age, gender, and duration were recorded for all participants. The fasting venous blood samples from all participants were analyzed for biochemical testing of serum glycated hemoglobin (HbA1C).

**Figure 1:**
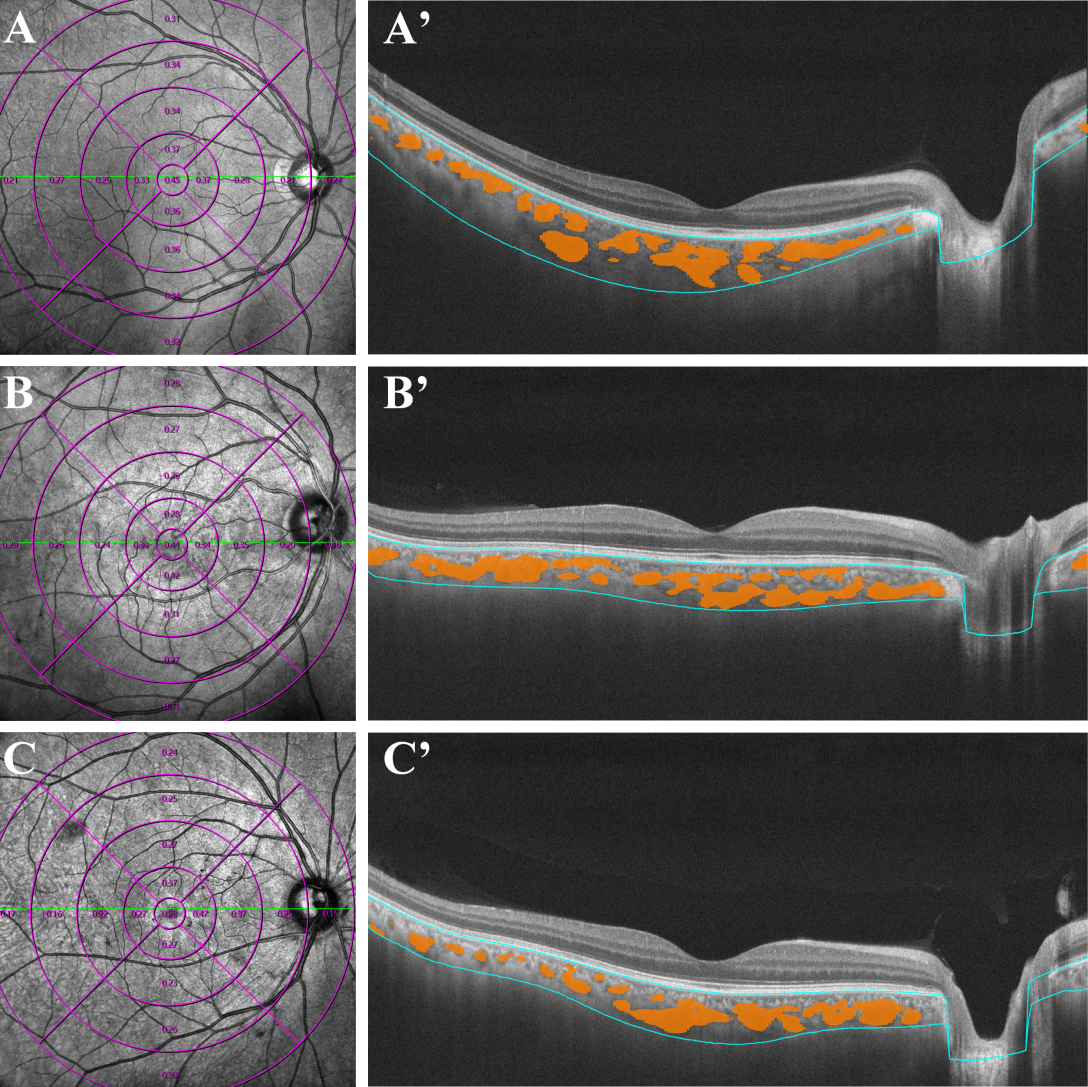
Examples of quantitative measurements of CVI using Wide-field OCTA in patients with early-stage DR (C, C'), diabetes mellitus without DR (B, B') and normal control subjects (A, A'). The left column consists of en face infrared (12 ×12 mm) images. The right column represent the optical coherence tomography of the retina and choroid across the fovea, yellow marks represent the choroidal lumen, and the blue lines indicate the upper and lower bounds of the choroid. CVI, choroidal vascularity index; OCTA, optical coherence tomographic angiography.

For OCTA examination, all patients were imaged using a commercial wide-field instrument (VG200, SVision Imaging, Ltd., Luoyang, China) with a 1,050 nm wavelength sweeping laser. All examinations were performed by an experienced ophthalmologist. The SS-OCTA scan was executed with a scanning speed of 200,000 A-scans per second and a wavelength-sweeping laser with a central wavelength of 1050nm. The axial resolution was 5 μm, the lateral resolution was 15 μm, and the scan depth was 3 mm. This equipment possessed eye-tracking technology involving an integrated confocal scanning laser ophthalmoscope to reduce eye-motion artifacts. Choroidal and retinal parameters acquired from wide-field SS-OCTA (including CVI, CT and CMT) were separately calculated in each annular region with built-in software of VG200, SVision Imaging (version 1.32.9.). We measured and automatically calculated the total choroidal area (TCA) and luminal area (LA) of the subjects under the calibration of a retinal specialist (Jianqiao Li). Choroidal vascular index (CVI) is defined as the ratio or proportion of the LA within the TCA (Shown in **Figure 1**), the ChT is defined as the vertical distance between the outer margin of the RPE-Bruch’s complex to the inner border of the choroidoscleral junction, and the CMT is defined as the mean distance in the central 12000-μm diameter area between the vitreoretinal interface and the outer margin of the RPE-Bruch’s complex. Besides, we also calculated the differentials of capillaries between different retinochoroid zones and the whole area (the values were calculated as Circle_x-y_ - average values, and the mean values of each group were shown in **Table 1**). All measurements were performed by the same ophthalmologist.

### Statistical analysis

All statistical analyses in this study were conducted using SPSS software (version 19.0 SPSS, Inc, Chicago, IL, USA). The Kolmogorov-Smirnov test was used for normality testing. Normally-distributed variables were expressed as the mean and standard deviation (SD), and non-parametric variables as the median and interquartile range (IQR). A paired-sample t-test was used to compare the number of patients enrolled, mean ages, durations, VA, Glucose, and Hemoglobin A1C between DM patients and healthy controls. Differences between the healthy controls, pre-DR, and early-stage DR groups were evaluated by generalized estimating equations (GEEs) for binocular data. The correlations between duration and choroidal characteristics were also calculated. A P-value < 0.05 was statistically significant.

## Results

### General clinical characteristics

A total of 131 eyes from 68 patients were included in the study, with no significant difference in age, sex composition or BCVA among groups (P_s_> 0.05). Significant differences were found between the pre-and early-stage DR groups in the duration of DM (9.02 ± 6.09 *vs*. 27.09 ± 13.51 (months)), blood glucose (9.05 ± 7.34 *vs*. 9.61 ± 8.47 (mmol/L)), and glycated hemoglobin (6.31 ± 4.25 *vs*. 8.62 ± 3.49 (%) (**Table 1**).

**Table 1:** Comparison of different retinal and choroidal zones' capillaries changes (Circle_x-y_ - Average ChT, CVI and CMT) in patients with early-stage diabetic retinopathy, DM without DR and non-diabetic individuals from the control group.

	Location	Normal control	DM without DR	p values	Early-stage DR	P values
**ChT**	Average thickness	287.48 ± 73.67	281.49 ± 50.65	N/A	270.41 ± 86.42	N/A
	Circle 0-3	38.16 ± 23.16	35.21 ± 25.18	p = 0.751	39.05 ± 30.05	p = 0.862
	Circle 3-6	26.47 ± 25.61	22.34 ± 23.82	p = 0.659	35.01 ± 31.28	p = 0.042*
	Circle 6-9	3.32 ± 20.45	-3.62 ± 22.09	p = 0.264	-5.68 ± 28.31	p = 0.083
	Circle 9-12	-20.21 ± 22.08	-26.30 ± 28.57	p = 0.182	-34.89 ± 27.65	p < 0.001*
**CVI**	Average CVI	0.292 ± 0.068	0.271 ± 0.064	N/A	0.240 ± 0.059	N/A
	Circle 0-3	0.052 ± 0.064	0.068 ± 0.071	p = 0.008*	0.069 ± 0.065	p = 0.007*
	Circle 3-6	0.028 ± 0.055	0.031 ± 0.059	p = 0.530	0.029 ± 0.059	p = 0.865
	Circle 6-9	-0.007 ± 0.061	0.006 ± 0.058	p = 0.027*	-0.009 ± 0.068	p = 0.721
	Circle 9-12	-0.019 ± 0.065	-0.031 ± 0.074	p = 0.032*	-0.038 ± 0.051	p = 0.003*
**CMT**	Average thickness	264.32 ± 9.45	261.09 ± 13.92	N/A	262.34 ± 10.56	N/A
	Circle 0-3	57.32 ± 11.59	52.08 ± 13.87	p = 0.772	59.66 ± 14.94	p = 0.914
	Circle 3-6	32.08 ± 12.07	28.04 ± 14.84	p = 0.801	26.47 ± 13.78	p = 0.710
	Circle 6-9	4.28 ± 11.67	2.51 ± 12.76	p = 0.482	2.69 ± 17.05	p = 0.517
	Circle 9-12	-9.88 ± 11.20	-11.29 ± 13.27	p = 0.207	-18.64 ± 19.57	p = 0.014*

Values were calculated as Circle_x-y_ - average values and shown as means ± SDs (). DM, diabetes mellitus; DR, diabetic retinopathy; ChT, Choroidal thickness; CVI, Choroidal Vascularity Index; CMT, central macular thickness. Statistical differences were analyzed between the groups of patients with DM and the normal control, p < 0.05 indicates a statistically significant difference, annotated with *. NA, not applicable.

### Characteristics of the choroid

**Figure 2:**
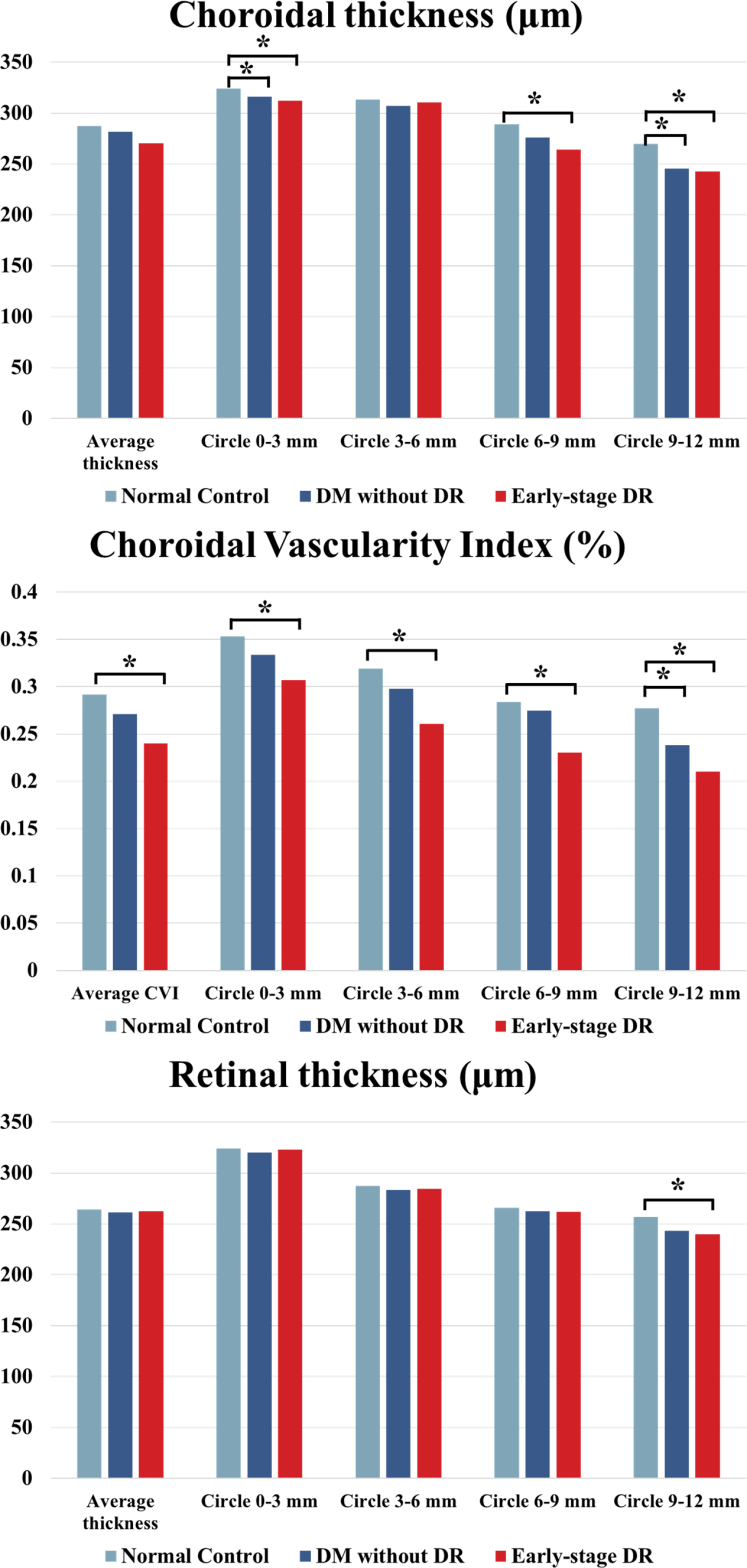
ChT, CVI and CMT comparison of patients with early-stage DR, diabetes mellitus without ophthalmoscopic signs of DR and normal control subjects of the control group. * indicates a statistically significant difference (p < 0.05).

The choroidal parameter values in each zone for the three groups are shown in **Table 2** and **Figure 2**. Although the mean CVI was lower in patients with pre- and early-stage DR than in healthy controls, a significant difference was only found for patients with early-stage DR (p = 0.013). During subgroup analyses, after stratifying by region, the differences in CVI values in the 9–12 mm range between the pre-DR group and the early-stage DR group decreased significantly compared to the control group (p = 0.016, p < 0.001, respectively). Moreover, ChT values were significantly reduced in the pre-DR group and the early-stage DR group in the 0–3 mm (p = 0.016, p = 0.002, respectively) and 9–12 mm ranges (p = 0.016, p = 0.002, respectively) and the early-stage DR group in the 6-9 mm range (p = 0.015). In addition, no changes were observed in any region except for a significant decrease in CMT in the 9–12 mm region of the early-stage DR group (p = 0.034).

**Table 2:** Demographic data of DM patients and healthy controls.

	Normal control	DM without DR	p values	Early-stage DR	P values
**Patients (Female)**	23 (12)	22 (12)	0.961	23(11)	1.000
**Age**	55.25 ± 9.65	57.34 ± 10.14	0.812	56.08 ± 12.07	0.873
**Eyes**	46	43	0.818	42	0.780
**Duration of DM (months)**	N/A	9.02 ± 6.09	N/A	27.09 ± 13.51	N/A
**Spherical equivalent (diopters)**	+0.67 ± 0.39	+0.52 ± 0.43	0.783	+0.58 ± 0.46	0.826
**VA (letters)**	82.09 ± 3.05	82.24 ± 2.02	0.971	82.43 ± 2.07	0.964
**GLU (mmol/L)**	N/A	9.05 ± 7.34	N/A	9.61 ± 8.47	N/A
**HbA1c (%)**	N/A	6.31 ± 4.25	N/A	8.62 ± 3.49	N/A

Values are presented as the means ± standard deviations at baseline in different groups. DM, diabetes mellitus; DR, diabetic retinopathy; VA, Visual acuity; in Early Treatment Diabetic Retinopathy Study (ETDRS) units; GLU, Glucose; HbA1c, Hemoglobin A1C. Statistical differences were analyzed between the patients with DM and the normal control, p < 0.05 indicates statistically significant difference. NA, not applicable.

**Figure 3:**
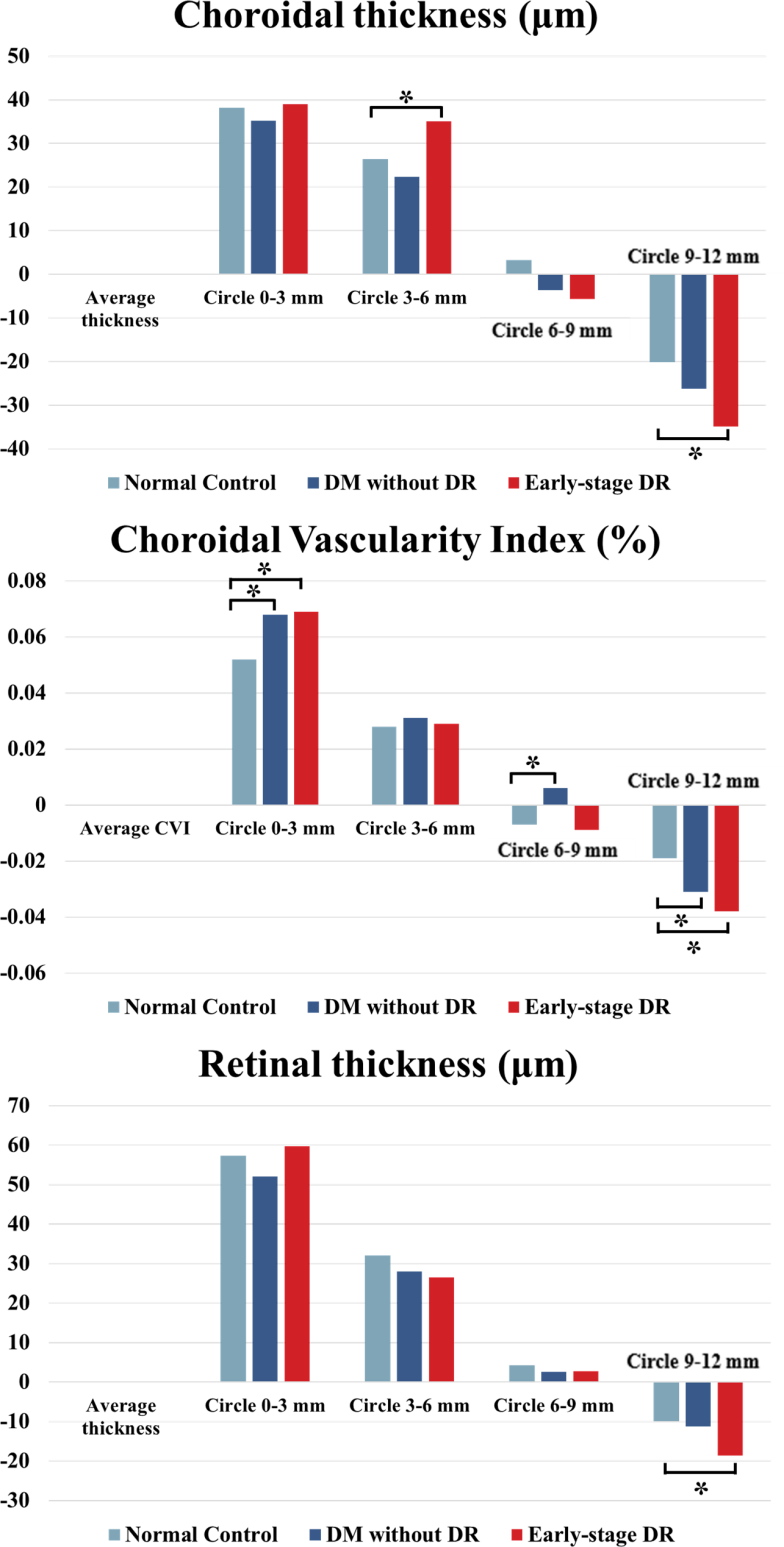
ChT, CVI and CMT changes in different fundus zones (Circle_x-y_ *vs*. Average density) in patients with early-stage DR, diabetes mellitus without ophthalmoscopic signs of DR and non-diabetic subjects of the control group. * indicates a statistically significant difference (p < 0.05).

The different parameters in different ranges and the whole area are shown in **Table 3** and **Figure 3**. Compared to each region of the control group, the changes in CVI values were significantly increased in the 0–3 mm, 6–9 mm, and 9–12 mm regions of the pre-DR patients (p = 0.008, p = 0.027, p = 0.032, respectively), and the CVI values were significantly increased in early-stage DR patients in the 0–3 mm and 9–12 mm regions(p = 0.007, p = 0.03, respectively). Besides, the ChT of early-stage DR patients was significantly decreased compared with healthy subjects in 3–6 mm and 9–12 mm zones (p = 0.042, p < 0.001, respectively). Finally, only the CMT in the 9–12 mm zone in the early-stage DR group decreased significantly compared with the healthy control group (p = 0.014).

**Table 3:** Optical coherence tomographical findings in patients with early-stage diabetic retinopathy, diabetes mellitus without DR and normal control group.

	Location	Normal control	DM without DR	p values	Early-stage DR	p values
**ChT**	Average thickness	287.48 ± 73.67	281.49 ± 50.65	p = 0.654	270.41 ± 86.42	p = 0.210
	Circle 0-3	324.26 ± 92.07	316.25 ± 71.63	p = 0.016*	312.35 ± 120.31	p = 0.002*
	Circle 3-6	313.27 ± 84.78	306.83 ± 62.24	p = 0.542	310.50 ± 107.11	p = 0.784
	Circle 6-9	289.05 ± 77.48	276.24 ± 51.58	p = 0.461	264.28 ± 84.03	p = 0.015*
	Circle 9-12	269.88 ± 65.64	245.37 ± 45.65	p = 0.008*	242.76 ± 75.93	p < 0.001*
**CVI**	Average CVI	0.292 ± 0.068	0.271 ± 0.064	p = 0.178	0.240 ± 0.059	p = 0.013*
	Circle 0-3	0.353 ± 0.120	0.334 ± 0.100	p = 0.097	0.307 ± 0.099	p = 0.020*
	Circle 3-6	0.319 ± 0.081	0.298 ± 0.080	p = 0.086	0.261 ± 0.075	p = 0.008*
	Circle 6-9	0.284 ± 0.063	0.275 ± 0.062	p = 0.310	0.230 ± 0.056	p = 0.007*
	Circle 9-12	0.277 ± 0.065	0.238 ± 0.062	p = 0.016*	0.210 ± 0.060	p < 0.001*
**CMT**	Average thickness	264.32 ± 9.45	261.09 ± 13.92	p = 0.863	262.34 ± 10.56	p = 0.716
	Circle 0-3	324.03 ± 14.26	320.01 ± 14.56	p = 0.841	323.05 ± 12.16	p = 0.890
	Circle 3-6	287.31 ± 11.28	283.17 ± 15.16	p = 0.806	284.64 ± 15.08	p = 0.773
	Circle 6-9	265.95 ± 10.16	262.47 ± 12.05	p = 0.754	261.87 ± 17.29	p = 0.681
	Circle 9-12	256.64 ± 10.08	243.12 ± 15.19	p = 0.069	240.08 ± 23.57	p = 0.034*

Values are showed as means ± SDs. DM, diabetes mellitus; DR, diabetic retinopathy; ChT, Choroidal thickness; CVI, Choroidal Vascularity Index; CMT, central macular thickness. Statistical differences were analyzed between the groups of patients with DM and the normal control, p < 0.05 indicates statistically significant difference, annotated with *.

The results of correlation analysis between the duration of DM and choroidal parameters are shown in **Table 4**. The disease duration in patients with DM was negatively correlated with ChT (p = 0.047, correlation coefficient= -0.395; p = 0.006, correlation coefficient= -0.549, respectively) and CVI (p = 0.038, correlation coefficient= -0.413; p = 0.004, correlation coefficient= -0.597, respectively) in 6–9 mm and 9–12mm ranges. Finally, the mean ChT negatively correlated with disease duration (p = 0.032, correlation coefficient= -0.420).

**Table 4:** The correlations between duration and choroidal characteristics in patients with diabetes mellitus.

	Characteristic	Mean values	Correlation coefficient	p values
**ChT**	Average thickness	276.32 ± 65.29	-0.420	p = 0.032*
	Circle 0-3	313.89 ± 92.05	-0.134	p = 0.236
	Circle 3-6	308.06 ± 88.04	-0.189	p = 0.125
	Circle 6-9	269.96 ± 74.23	-0.395	p = 0.047*
	Circle 9-12	243.86 ± 61.98	-0.549	p = 0.006*
**CVI**	Average CVI	0.257 ± 0.063	-0.178	p = 0.146
	Circle 0-3	0.321 ± 0.097	-0.057	p = 0.463
	Circle 3-6	0.282 ± 0.083	-0.128	p = 0.216
	Circle 6-9	0.254 ± 0.057	-0.413	p = 0.038*
	Circle 9-12	0.225 ± 0.063	-0.597	p = 0.004*

Values are shown as means ± SDs. ChT, Choroidal thickness; CVI, Choroidal Vascularity Index; p < 0.05 indicates a statistically significant difference, annotated with *.

## Discussion

In this cross-sectional study, wide-field SS-OCTA examinations were performed on three groups (healthy subjects and patients with pre and early-stage DR). To investigate the early changes of choroidal vascular structure in the hyperglycemia environment and to clarify the pathological features of diabetic retinopathy, we assessed CVI, CMT, and ChT of the retinal and choroidal capillaries based on a 12 × 12 mm field of view, as well as the correlations between DM duration and retinochoroid parameters. The CVI and ChT values of DR patients were significantly lower than the healthy control group, and the CVI and ChT values in peripheral areas of DM patients decreased more significantly than those in central regions. However, the CMT outside the 9-12 mm range did not decrease significantly in most areas in DR patients.

Moreover, there was a significant decrease in CVI between the two DM groups and the control group (p = 0.035), suggesting that the choroidal perfusion might decrease in DM patients even before the onset of macroscopic retinochoroid lesions. Tan et al. reported that the CVI of the DM group was significantly lower than the control group, while the CVI of the DR group was lower than the non-DR group, indicating that the choroidal capillaries in diabetic patients decreased significantly (27), consistent with the present study findings. Another study by Foo, V. H. et al. substantiated that the CVI of Haller’s layer in the eyes of pre-DR patients was significantly lower than in healthy controls, suggesting that early diabetic choroidal damages could affect larger choroidal capillaries much earlier (28). Unlike the past studies, the scanning range of wide-field SS-OCTA was expanded to 12 × 12mm with the fovea of the macula as the center in the present study, which enables observation of the microcirculation of the peripheral retinochoroid capillaries.

To further explore the structural changes of choroidal capillaries in different areas, the wide-field OCTA images of the three groups were divided into four concentric circles according to the distance from the fovea. The decreases in CVI values within the 9–12 mm circle region were most significant in four concentric circles of the early-stage DR and pre-DR groups, suggesting that the choroidal circulation of peripheral retinochoroid areas was more sensitive to blood glucose changes than the central areas. It is reasonable to speculate that disease progression may begin in peripheral areas, and the choroidal capillaries may be the most seriously affected area. Indeed, the scanning range of OCTA devices was limited to 6 × 6mm in most previous studies, and the wide-field OCTA adopted in our study makes up SS-OCTA makes up for the narrow visual field of traditional OCTA.

An increasing body of evidence suggests that ChT can quantify the structural changes of the choroid. Nonetheless, no consensus has been reached on the changes in ChT in DM patients (29, 30). Querques, G. et al. indicated that ChT of DR patients might decrease (31); however, Kim, J.T. et al. found that the choroidal thickness decreased in the early stages of DR but increased in the stage of severe NPDR or PDR (32). DM is a metabolic disease affecting the retinal and choroidal vasculature. Although the principal changes in diabetic eyes occur in the retinal vasculature, additional changes have been paid more and more attention in recent years. Histologic studies showed increased tortuosity, and the formation of sinuslike structures between the choroidal lobules, what’s more, in some advanced cases, luminal narrowing of the capillaries, capillary dropout, and focal scarring were also observed (33). In our study, the ChT between the DR group in the range of 0–3/6–9/9–12 mm decreased significantly (P<0.05) compared with the control group. In addition, the decrease in ChT was more significant in the peripheral area. However, there was no significant difference in CMT between DM patients without DR and healthy control, while only a slight decrease was observed in the peripheral areas of early-stage DR patients. However, the positive result was somewhat accidental in the analysis of CMT. Although there was no significant change in retinal thickness, the choroidal thickness decreased significantly. It can be concluded that CMT is insensitive in the early stage of DR and poorly reflects early damage to the retinal capillaries. Accordingly, choroidal parameters may be more sensitive indicators for patients with pre- and early-stage DR.

In addition, correlation analysis between CVI, ChT and the duration of DR showed a significant negative correlation between CVI and ChT at 6–9 mm and 9–12mm (P<0.05), and a significant correlation was observed at 9–12 mm (P<0.01). A longer DR duration was associated with lower choroidal CVI and ChT values. During the early diagnosis of DR, peripheral CVI and ChT can be used as evaluation indexes, indicating choroidal damage in DM patients without visible pathological retina changes more sensitively. Therefore, wide-field SS-OCTA is a more sensitive and effective tool to evaluate disease progression in DM patients.

Some limitations and shortcomings were present in the current research. First of all, the sample size was small. Indeed, a larger sample size is required in future studies to obtain more reliable and robust results. Besides, patients with proliferative DR should be enrolled to study the relationship between CVI changes and the different stages of DR. Moreover, the cross-section areas studied in the present study do not enable dynamic evaluation of the choroid changes with disease progression. Further longitudinal prospective research is warranted to confirm current results. Finally, hypertension and functional impairment are common in patients with diabetes (34, 35), which may have an impact on choroidal blood flow, but we did not make a detailed analysis due to the lack of relevant data.

In conclusion, we comprehensively used wide-field SS-OCTA to explore the characteristics of choroidal microcirculation structure in DM patients. The results showed that CVI and ChT decreased significantly, especially in the peripheral area (9-12 mm) in the pre-and early-stage DR groups, which indicates that monitoring of choroidal microcirculation parameters is essential for early diagnosis of DR.

## Data availability statement

The raw data supporting the conclusions of this article will be made available by the authors, without undue reservation.

## Ethics statement

The studies involving human participants were reviewed and approved by Medical Research and Ethics Committee of Qilu Hospital. The patients/participants provided their written informed consent to participate in this study.

## Author contributions

(I) Conception and design: ZnL, FX and JL. (II) Administrative support: SW and JL. (III) Provision of study materials or patients: FX, ZnL and XY. (IV) Collection and assembly of data: ZnL, XY, ZiL and GL. (V) Data analysis and interpretation: FX and ZnL. All authors contributed to the article and approved the submitted version.

## References

[1] Collaboration NCDRF. Worldwide trends in diabetes since 1980: a pooled analysis of 751 population-based studies with 4.4 million participants. Lancet (2016) 387(10027):1513–30. doi: 10.1016/S0140-6736(16)00618-8 PMC5081106 27061677

[2] Danaei G Finucane MM Lu Y Singh GM Cowan MJ Paciorek CJ. National, regional, and global trends in fasting plasma glucose and diabetes prevalence since 1980: systematic analysis of health examination surveys and epidemiological studies with 370 country-years and 2.7 million participants. Lancet (2011) 378(9785):31–40. doi: 10.1016/S0140-6736(11)60679-X 21705069

[3] Wong TY Cheung CM Larsen M Sharma S Simo R. Diabetic retinopathy. Nat Rev Dis Primers (2016) 2:16012. doi: 10.1038/nrdp.2016.12 27159554

[4] Kastelan S Oreskovic I Biscan F Kastelan H Gverovic Antunica A. Inflammatory and angiogenic biomarkers in diabetic retinopathy. Biochem Med (Zagreb) (2020) 30(3):30502. doi: 10.11613/BM.2020.030502 PMC7394255 32774120

[5] Zhang Y Xu F Lin Z Wang J Huang C Wei M. Prediction of visual acuity after anti-VEGF therapy in diabetic macular edema by machine learning. J Diabetes Res (2022) 2022:5779210. doi: 10.1155/2022/5779210 35493607 PMC9042629

[6] Rubsam A Parikh S Fort PE. Role of inflammation in diabetic retinopathy. Int J Mol Sci (2018) 19(4). doi: 10.3390/ijms19040942 PMC5979417 29565290

[7] Nguyen TT Wong TY. Retinal vascular changes and diabetic retinopathy. Curr Diabetes Rep (2009) 9(4):277–83. doi: 10.1007/s11892-009-0043-4 19640340

[8] Durbin MK An L Shemonski ND Soares M Santos T Lopes M. Quantification of retinal microvascular density in optical coherence tomographic angiography images in diabetic retinopathy. JAMA Ophthalmol (2017) 135(4):370–6. doi: 10.1001/jamaophthalmol.2017.0080 PMC5470403 28301651

[9] Mendrinos E Stangos AN Pournaras CJ. Diabetic retinopathy. BMJ Clin Evid (2007) 2007. PMC2943811 19450351

[10] Wang XN Li ST Li W Hua YJ Wu Q. The thickness and volume of the choroid, outer retinal layers and retinal pigment epithelium layer changes in patients with diabetic retinopathy. Int J Ophthalmol (2018) 11(12):1957–62. doi: 10.18240/ijo.2018.12.14 PMC6288528 30588430

[11] Alves CH Fernandes R Santiago AR Ambrosio AF. Microglia contribution to the regulation of the retinal and choroidal vasculature in age-related macular degeneration. Cells (2020) . doi: 10.3390/cells9051217 PMC7290930 32423062

[12] Lu ES Cui Y Le R Zhu Y Wang JC Lains I. Detection of neovascularisation in the vitreoretinal interface slab using widefield swept-source optical coherence tomography angiography in diabetic retinopathy. Br J Ophthalmol Apr (2022) 106(4):534–9. doi: 10.1136/bjophthalmol-2020-317983 PMC9092312 33355148

[13] Bandello F Corbelli E Carnevali A Pierro L Querques G. Optical coherence tomography angiography of diabetic retinopathy. Dev Ophthalmol (2016) 56:107–12. doi: 10.1159/000442801 27023453

[14] Saracco JB Gastaud P Ridings B Ubaud CA. [Preliminary study on diabetic choroidopathy]. Bull Soc Ophtalmol Fr (1982) 82(3):451–4. Etude preliminaire sur la choroidopathie diabetique. 7116547

[15] Lutty GA. Diabetic choroidopathy. Vision Res Oct (2017) 139:161–7. doi: 10.1016/j.visres.2017.04.011 PMC5858724 28535994

[16] Wang JC Lains I Providencia J Armstrong GW Santos AR Gil P. Diabetic choroidopathy: Choroidal vascular density and volume in diabetic retinopathy with swept-source optical coherence tomography. Am J Ophthalmol (2017) 184:75–83. doi: 10.1016/j.ajo.2017.09.030 28988899

[17] Kim M Ha MJ Choi SY Park YH. Choroidal vascularity index in type-2 diabetes analyzed by swept-source optical coherence tomography. Sci Rep (2018) 8(1):70. doi: 10.1038/s41598-017-18511-7 29311618 PMC5758605

[18] Hamadneh T Aftab S Sherali N Vetrivel Suresh R Tsouklidis N An M. Choroidal changes in diabetic patients with different stages of diabetic retinopathy. Cureus (2020) 12(10):e10871. doi: 10.7759/cureus.10871 33178524 PMC7652371

[19] Hormel TT Hwang TS Bailey ST Wilson DJ Huang D Jia Y. Artificial intelligence in OCT angiography. Prog Retin Eye Res (2021) 85:100965. doi: 10.1016/j.preteyeres.2021.100965 33766775 PMC8455727

[20] Hormel TT Jia Y Jian Y Hwang TS Bailey ST Pennesi ME. Plexus-specific retinal vascular anatomy and pathologies as seen by projection-resolved optical coherence tomographic angiography. Prog Retin Eye Res (2021) 80:100878. doi: 10.1016/j.preteyeres.2020.100878 32712135 PMC7855241

[21] Safi H Safi S Hafezi-Moghadam A Ahmadieh H. Early detection of diabetic retinopathy. Surv Ophthalmol (2018) 63(5):601–8. doi: 10.1016/j.survophthal.2018.04.003 29679616

[22] Simonett JM Scarinci F Picconi F Giorno P De Geronimo D Di Renzo A. Early microvascular retinal changes in optical coherence tomography angiography in patients with type 1 diabetes mellitus. Acta Ophthalmol (2017) 95(8):e751–5. doi: 10.1111/aos.13404 28211261

[23] Ting DSW Tan GSW Agrawal R Yanagi Y Sie NM Wong CW. Optical coherence tomographic angiography in type 2 diabetes and diabetic retinopathy. JAMA Ophthalmol (2017) 135(4):306–12. doi: 10.1001/jamaophthalmol.2016.5877 28208170

[24] Soares M Neves C Marques IP Pires I Schwartz C Costa MA. Comparison of diabetic retinopathy classification using fluorescein angiography and optical coherence tomography angiography. Br J Ophthalmol (2017) 101(1):62–8. doi: 10.1136/bjophthalmol-2016-309424 27927677

[25] Pichi F Smith SD Abboud EB Neri P Woodstock E Hay S. Wide-field optical coherence tomography angiography for the detection of proliferative diabetic retinopathy. Graefes Arch Clin Exp Ophthalmol (2020) 258(9):1901–9. doi: 10.1007/s00417-020-04773-x 32474692

[26] Qian Y Yang J Liang A Zhao C Gao F Zhang M. Widefield swept-source optical coherence tomography angiography assessment of choroidal changes in vogt-Koyanagi-Harada disease. Front Med (Lausanne) (2021) 8:698644. doi: 10.3389/fmed.2021.698644 34604253 PMC8481635

[27] Tan KA Laude A Yip V Loo E Wong EP Agrawal R. Choroidal vascularity index - a novel optical coherence tomography parameter for disease monitoring in diabetes mellitus? Acta Ophthalmol (2016) 94(7):e612–6. doi: 10.1111/aos.13044 27151819

[28] Foo VHX Gupta P Nguyen QD Chong CCY Agrawal R Cheng CY. Decrease in choroidal vascularity index of haller's layer in diabetic eyes precedes retinopathy. BMJ Open Diabetes Res Care (2020) 8(1). doi: 10.1136/bmjdrc-2020-001295 PMC7482468 32912848

[29] Lains I Talcott KE Santos AR Marques JH Gil P Gil J. Choroidal thickness in diabetic retinopathy assessed with swept-source optical coherence tomography. Retina (2018) 38(1):173–82. doi: 10.1097/IAE.0000000000001516 28196053

[30] Tavares Ferreira J Vicente A Proenca R Santos BO Cunha JP Alves M. Choroidal thickness in diabetic patients without diabetic retinopathy. Retina (2018) 38(4):795–804. doi: 10.1097/IAE.0000000000001582 28267113

[31] Querques G Lattanzio R Querques L Del Turco C Forte R Pierro L. Enhanced depth imaging optical coherence tomography in type 2 diabetes. Invest Ophthalmol Vis Sci (2012) 53(10):6017–24. doi: 10.1167/iovs.12-9692 22879414

[32] Kim JT Lee DH Joe SG Kim JG Yoon YH. Changes in choroidal thickness in relation to the severity of retinopathy and macular edema in type 2 diabetic patients. Invest Ophthalmol Vis Sci (2013) 54(5):3378–84. doi: 10.1167/iovs.12-11503 23611988

[33] Hidayat AA Fine BS. Diabetic choroidopathy. light and electron microscopic observations of seven cases. Ophthalmology (1985) 92(4):512–22. doi: 10.1016/S0161-6420(85)34013-7 2582331

[34] Peng Q Hu Y Huang M Wu Y Zhong P Dong X. Retinal neurovascular impairment in patients with essential hypertension: An optical coherence tomography angiography study. Invest Ophthalmol Vis Sci (2020) 61(8):42. doi: 10.1167/iovs.61.8.42 PMC7425736 32725211

[35] Zeng X Hu Y Chen Y Lin Z Liang Y Liu B. Retinal neurovascular impairment in non-diabetic and non-dialytic chronic kidney disease patients. Front Neurosci (2021) 15:703898. doi: 10.3389/fnins.2021.703898 34867144 PMC8639216

